# *Saccharomyces* genome database provides new regulation data

**DOI:** 10.1093/nar/gkt1158

**Published:** 2013-11-20

**Authors:** Maria C. Costanzo, Stacia R. Engel, Edith D. Wong, Paul Lloyd, Kalpana Karra, Esther T. Chan, Shuai Weng, Kelley M. Paskov, Greg R. Roe, Gail Binkley, Benjamin C. Hitz, J. Michael Cherry

**Affiliations:** Department of Genetics, Stanford University School of Medicine, Stanford, CA 94305, USA

## Abstract

The *Saccharomyces* Genome Database (SGD; http://www.yeastgenome.org) is the community resource for genomic, gene and protein information about the budding yeast *Saccharomyces cerevisiae,* containing a variety of functional information about each yeast gene and gene product. We have recently added regulatory information to SGD and present it on a new tabbed section of the Locus Summary entitled ‘Regulation’. We are compiling transcriptional regulator–target gene relationships, which are curated from the literature at SGD or imported, with permission, from the YEASTRACT database. For nearly every *S. cerevisiae* gene, the Regulation page displays a table of annotations showing the regulators of that gene, and a graphical visualization of its regulatory network. For genes whose products act as transcription factors, the Regulation page also shows a table of their target genes, accompanied by a Gene Ontology enrichment analysis of the biological processes in which those genes participate. We additionally synthesize information from the literature for each transcription factor in a free-text Regulation Summary, and provide other information relevant to its regulatory function, such as DNA binding site motifs and protein domains. All of the regulation data are available for querying, analysis and download via YeastMine, the InterMine-based data warehouse system in use at SGD.

## INTRODUCTION

The *Saccharomyces* Genome Database [SGD; http://www.yeastgenome.org; (1)] provides functional information about *Saccharomyces cerevisiae* genes and proteins in the form of Gene Ontology (GO) annotations (2), mutant phenotype annotations and free-text Descriptions. If a gene product participates in a regulatory process, this is noted both in its GO annotations and in its Description. However, the targets of each regulator have not, until now, been captured systematically in a controlled format.

A complete understanding of the regulatory networks in a cell requires a comprehensive catalog of regulatory interactions in a machine-readable format that can be used for computation and analysis. Creating such a catalog is an ambitious goal because of the multiple levels on which regulation can occur.

To address this goal, we have begun to curate information about transcriptional regulation, one of the best-studied regulatory mechanisms in budding yeast. Combining regulation annotations generated by SGD and the YEASTRACT database [http://yeastract.com/; (3)] has provided full-genome coverage of transcriptional regulatory data. We have added high-confidence regulatory data for nearly all *S. cerevisiae* genes on a new gene-specific Regulation page that displays a table of transcriptional regulators for each gene. For genes that encode transcriptional regulators, the Regulation page displays additional data relevant to their regulatory roles. We are also adding free-text paragraphs summarizing the regulatory role of each transcription factor (TF), starting with a set of 147 TFs that bind DNA directly. We describe here the scope and origin of regulation data currently in SGD and how to access them.

## RESULTS

### Transcription factors in *S. cerevisiae*

Various estimates have put the number of TFs encoded by the yeast genome at ∼140–250 [reviewed in (4,5)]. The number varies according to the criteria used to classify proteins as TFs: some studies count proteins with predicted DNA-binding domains; others include only proteins shown to bind DNA directly; still others include non-DNA binding subunits of transcription factor complexes.

We decided to compile a working set of manageable size for deeper curation in this first phase of the project by focusing on TFs that bind DNA directly. To assemble this set, we started with the ‘expert curated’ set of TFs (209 TFs) from the Yeast Transcription Factor Specificity Compendium [YeTFaSCo; http://yetfasco.ccbr.utoronto.ca/; (6)]. We compared this list with the 163 *S. cerevisiae* TFs classified in the JASPAR database [http://jaspar.genereg.net/; (7)] and also to the list of genes having GO annotations in SGD that reflected DNA-binding TF activity (annotated with the Molecular Function term GO:0003700, ‘sequence-specific DNA binding transcription factor activity’, or related terms). We added genes present on all three of these lists to our working set. When a gene was present on some but not all of the lists, we examined the published evidence and in some cases added these genes to the set. The end result of this analysis was a set of 147 TFs on which we focused our efforts to write Regulation Summary paragraphs.

### Discovering regulatory relationships

When the product of one gene (a regulator) regulates the transcription of another gene (a target), the two genes are said to have a regulatory relationship. Regulatory relationships can be inferred from several different kinds of experiment, both large- and small-scale.

Early research into mechanisms of transcriptional regulation used small-scale approaches to establish that individual TFs bind promoter regions and activate or repress transcription. Techniques such as DNase footprinting or electrophoretic mobility shift assay can delineate TF binding sites, whereas TF activity can be measured *in vivo* or *in vitro* by looking at expression of a reporter gene under control of a target gene promoter. Small-scale experiments continue to provide ‘gold-standard’ data on transcriptional regulation.

Large-scale experiments generate huge amounts of genome-wide regulation data using chromatin immunoprecipitation-based methods [ChIP-chip, ChIP-seq, ChIP-exo; reviewed in (5)] to locate the direct genomic binding sites of a particular TF. Localization of a TF to the promoter region of a gene strongly suggests that the gene is a direct target of regulation by the TF. These data are often accompanied by a measure of statistical significance, such as a *P*-value, which informs on the probability that TF binding is specific at a particular site; however, both false positives and false negatives may occur. Binding per se does not reveal whether the TF activates or represses transcription of the target gene.

Another commonly used large-scale method for determining regulatory relationships is the generation of genome-wide expression data, using microarrays or RNA-seq, in a strain where the gene for a particular TF has been deleted, otherwise mutated or overexpressed [reviewed in (5)]. A change in transcription of a gene in the TF mutant strain relative to wild-type implies that the gene may be a target of that TF and establishes the positive or negative nature of its regulation. However, this method does not distinguish between direct and indirect regulation: a change in the expression of a target gene could be the direct effect of the TF mutation or it could represent the indirect effect of the TF mutation on another regulator.

The highest-confidence regulatory relationships are supported by both binding and expression evidence. However, at present, these relationships represent a small proportion of the total data (5,8). Re-analysis of existing binding and expression data can provide confidence measures for known relationships or lead to the discovery of new relationships. Researchers are actively examining ways to improve computational methods for this kind of re-analysis (8–10).

An important point to consider when interpreting regulatory relationship data is that relationships are dependent both on environmental conditions and on genetic background. Only a small number of TFs are active under standard growth conditions (rich glucose-based medium, 30°C), with most functioning in the response to specific stresses or environments, so it is essential to gather data under a variety of conditions (11). Differential expression analysis also reveals transcriptional variation under different growth conditions: in *S. cerevisiae* expression of at least 60% of genes is enriched 2-fold under at least one non-standard growth condition (12). The genetic background of the experimental strain is also a relevant factor. For example, Zheng *et al.* (13) found significant variation between two common laboratory strains in the genomic locations where the TF Ste12p was bound; most of the variation could be ascribed to polymorphisms in its binding sites.

### Regulatory relationship data in SGD

Regulatory relationship data in SGD are curated from the types of experiment described previously that provide evidence for transcriptional regulation of target genes by TFs. These data are currently derived from two sources. SGD biocurators have curated recent large-scale studies providing regulation data and will continue this on an ongoing basis as they are published. These data are complemented by data from the YEASTRACT database, which curates regulatory relationships from a wide range of publications including both high-throughput and small-scale studies [http://yeastract.com/; (3)]. To provide SGD users with the ability to access and analyze the maximum amount of regulation data, we initiated a collaboration with the YEASTRACT group and received permission to incorporate YEASTRACT regulatory data into SGD; we will continue to import future data releases. To avoid duplicate data rows, when both groups have curated the same article we filter out the YEASTRACT annotations and display only the SGD annotations.

Each regulation annotation comprises a regulator gene, a target gene and supporting information. The types of supporting data that are included in regulation annotations in SGD are shown in Table 1. Because each experimental method for studying regulatory relationships has its own particular advantages and disadvantages, we note the method used to determine each relationship using the Evidence Code Ontology (ECO; http://evidenceontology.org). The YEASTRACT project uses an internal set of evidence codes, and we have mapped these to ECO terms for display at SGD.

**Table 1.** Types of data accompanying regulatory annotations in SGD

Data type	Description	Value	Additional notes
Regulator gene	Gene identifier	Genetic name and systematic name	
Target gene	Gene identifier	Genetic name and systematic name	
Evidence	ECO term	ECO term name and identifier	YEASTRACT-specific evidence codes are mapped to ECO by SGD
Condition	Experimental condition such as temperature, growth phase, growth medium and chemical stress	Free text	Noted for some but not all annotations
Direction	Activation versus repression	‘activated’ or ‘repressed’	Noted where available; YEASTRACT ‘positive’ or ‘negative’ are mapped to ‘activated’ or ‘repressed’ by SGD
Strain	Genetic background of the *S. cerevisiae* strain used in the experiment	Strain background name	Noted for SGD-curated annotations
Confidence score	Measure of the probability that a binding site is not due to chance	*P*-value or false discovery rate (FDR)	Noted for SGD-curated annotations where available
Source	Database project by which the curation was performed	SGD or YEASTRACT	
Reference	Published article supporting the regulatory relationship	PubMed ID	

As discussed previously, the condition under which an experiment was performed is also critical for data interpretation. Both SGD and YEASTRACT have captured condition information for experiments where available. At present, this information is noted in free text format by both databases. Where possible, both databases also record the direction of regulation. All SGD annotations include the genetic background of the yeast strain used in the experiment. Strain background names are derived from the controlled-vocabulary list that is used for mutant phenotype curation at SGD (14). For experiments whose data are associated with a confidence score such as *P*-value or false discovery rate, SGD annotations also include those scores.

### The regulation tab page

As of October 2013, 99% of all protein-coding genes in the S288C reference genome (5777 of 5820 verified and uncharacterized open reading frames) were represented in the regulatory relationship data in SGD as targets of regulation, and 401 of these were also represented in the data as regulators. Additionally, target genes included tRNAs, snoRNAs and other RNAs, as well as some dubious ORFs. ORFs are classified as dubious at SGD if they are judged unlikely to encode expressed proteins (15); however, data exist for these chromosomal regions because the corresponding probes are often present on microarrays, and SGD collects data for these ORFs in case evidence for their biological relevance becomes available in the future.

**Figure 1.**
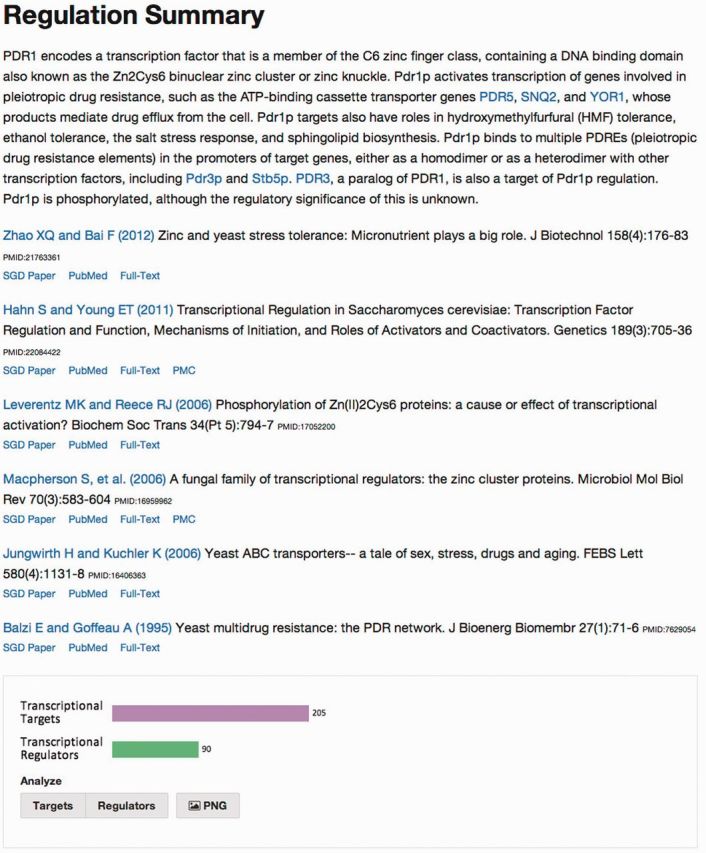
Summary features of the Regulation page. Top, the Regulation Summary paragraph, written by SGD biocurators, provides an overview of the context in which a TF acts. The Regulation Summary paragraph for the TF Pdr1p is shown. Gene and protein names in the Regulation Summary paragraph are linked to their SGD Locus Summary pages, and reference citations are linked to their SGD Paper pages. The Regulation Summary appears only on the Regulation pages for TFs. Bottom, a graphic indicates the number of targets and regulators for the gene of the page. This summary graphic is present on Regulation pages for all genes. The ‘Targets’ and ‘Regulators’ buttons generate lists of the targets or the regulators and their Descriptions and present links to download the gene list or to analyze it further by sending it to the GO Term Finder [http://www.yeastgenome.org/cgi-bin/GO/goTermFinder.pl; (21)], GO Slim Mapper http://www.yeastgenome.org/cgi-bin/GO/goSlimMapper.pl), SPELL [http://spell.yeastgenome.org/; (22)] or YeastMine [http://yeastmine.yeastgenome.org/yeastmine/begin.do; (23)] tools. The graphic may also be captured as an image file.

**Figure 2.**
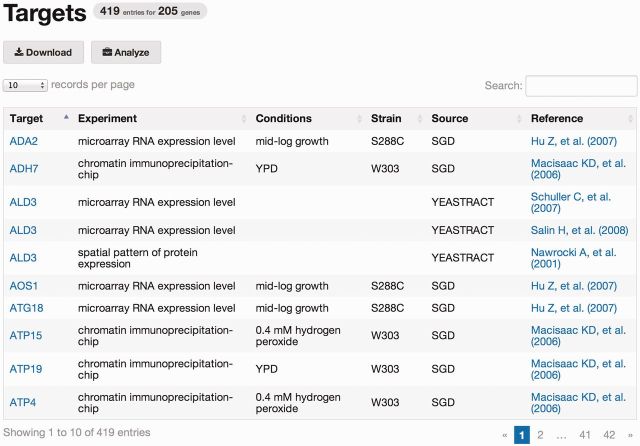
The Targets table for an example regulator gene, *PDR1*. The table header shows the number of annotations in the table as well as the number of unique target genes. Data types displayed in the table are described in Table 1. Columns may be sorted by clicking the up or down arrows in the column headers. The ‘Search’ box filters the table so that only those rows containing the search criteria are displayed. The ‘Download’ button downloads table data as a tab-delimited text file. The ‘Analyze’ button generates a list of the genes in the table, with their Descriptions, that may be downloaded or analyzed further using the GO Term Finder [http://www.yeastgenome.org/cgi-bin/GO/goTermFinder.pl; (21)], GO Slim Mapper (http://www.yeastgenome.org/cgi-bin/GO/goSlimMapper.pl), SPELL [http://spell.yeastgenome.org/; (22)] or YeastMine [http://yeastmine.yeastgenome.org/yeastmine/begin.do; (23)] tools. The Regulators table, showing regulators whose target is the gene of the page, shares the same interactive features.

**Figure 3.**
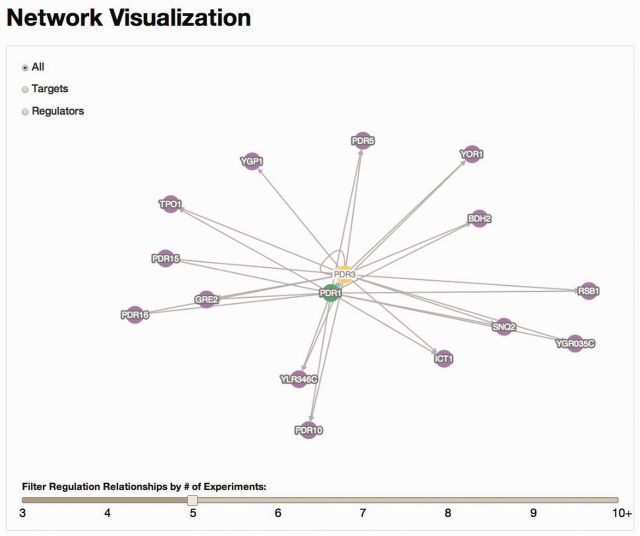
Network visualization for an example gene, *PDR3*, showing regulatory relationships supported by five or more annotations. The network is drawn using Cytoscape.js (http://cytoscape.github.io/cytoscape.js/; (24)]. Users can zoom in and out of the network view using a trackpad or mouse wheel and reposition genes by dragging. Genes depicted in the graphic are color coded by their relationships with the central gene: regulators are green, whereas targets are purple. Radio buttons in the top left corner allow display of only targets or only regulators, and the adjustable slider at the bottom sets the minimum number of annotations supporting the relationships shown. To present meaningful networks, we limit each to no >100 genes and no >250 regulatory relationships. A network visualization does not appear for genes whose regulatory relationship data do not meet these criteria: for example, a gene that has >250 regulatory relationships, each supported by just one annotation.

The types of information present on the Regulation tab pages, and their sources, are described later in the text in the order in which they appear on the pages. All of the tables and graphics on the pages have interactive features (see Figures 1–3).

#### Regulation summary paragraphs

For each of the 147 TFs in our initial set, SGD biocurators have surveyed the literature to write a free-text Regulation Summary paragraph that gives a capsule summary of the place of that TF in the regulatory network (Figure 1). The paragraph may include the protein family classification of the TF, its target genes and processes and information about the regulation of expression and activity of the TF itself. Because it is intended to be a brief overview, the citations for this paragraph are not comprehensive and review articles are cited wherever possible.

#### Target and regulator summary graphic

Directly below the Regulation Summary paragraph (or at the top of the Regulation page, for genes without paragraphs) is a graphic summarizing the numbers of targets and regulators for the gene, reflecting the number of other genes with which the gene of the page has regulator or target relationships (Figure 1).

#### Structural and domain information

Because most TFs belong to distinct families and have characteristic domains involved in DNA binding and interaction with the transcription machinery, we display domain and family information for all genes that are currently present as regulators in the regulator–target relationship data. The Domains and Classification table lists the domains found by InterProScan analysis (16) using the protein sequence of the regulator to query for domains and motifs from several databases. For each domain, amino acid coordinates are shown, along with its accession ID, its description and the source of the domain information. The accession ID is linked to the entry for that domain in its source database. This table also displays the class and family membership of the TF, as defined by the JASPAR database [(http://jaspar.genereg.net/; (7)]. The accession ID is linked to the summary page for that TF in the JASPAR database.

#### DNA binding site sequences and locations

The DNA Binding Site Motifs section of the Regulation page displays high-confidence binding site sequence motif(s) from the YeTFaSCo database [http://yetfasco.ccbr.utoronto.ca/; (6)]. In the sequence logos, each of the four nucleotides is represented using a different color, and the height of the letter representing a nucleotide is proportional to the frequency with which that nucleotide occurs at that position (17). Each logo image is linked to its YeTFaSCo record. These logos are displayed for all regulators having a high-confidence binding motif in YeTFaSCo.

The genomic locations of matches to these motifs are also available for viewing as a track in SGD’s genome browser [http://browse.yeastgenome.org; (18)]. To generate this track, position-weighted matrices [PWMs; (19)] were derived from the ‘expert curation’ set available at YeTFaSCo by filtering for high and medium confidence motifs. The Find Individual Motif Occurrences (FIMO) algorithm (20) was then used to determine the PWM-predicted binding site matches in the *S. cerevisiae* strain S288C reference genome, using the default parameters, a background of A,T: 0.31, G:C: 0.19 and a *P*-value threshold of 0.0001.

The ‘View predicted binding sites in GBrowse’ link on the Regulation page for a TF leads to a search of genome browser data for all PWM-predicted binding sites for that TF, including multiple motifs if there is more than one high-confidence binding motif. On the GBrowse search results page, a graphic represents the chromosomal locations of the motif matches, and the matches are listed with their coordinates. Each listing is linked to a GBrowse view of the chromosomal region where the match is located.

#### Table of transcriptional regulatory targets

The Regulation tab page for each regulator gene contains a ‘Targets’ table containing regulation annotations supporting its relationships with its target genes (Figure 2). As of October 2013, 401 genes were identified as regulators in the pool of regulator-target data. This includes the 147 DNA-binding TFs selected for deeper curation at SGD, as well as other transcriptional regulators that are represented in the regulation annotations generated both by SGD and YEASTRACT.

The Targets table displays most of the data types that comprise a regulation annotation (see Table 1), with the exception of regulation direction and confidence score. The complete annotations, including those data, are available for download via YeastMine [(23); see later in the text].

#### GO enrichment of targets

Knowledge of the processes or pathways in which a group of target genes is involved can help illuminate the biological role of their regulators. To identify the processes shared among the targets of a particular regulator, each set of targets is analyzed using the GO enrichment function of YeastMine (23). When Biological Process GO terms are significantly overrepresented among the target set of genes, these terms are displayed in the Shared GO Processes table. For each GO term, the number of target genes annotated with that term and the significance of the enrichment (*P*-value) are shown. The Holm–Bonferroni correction is used in the calculation, and terms that are enriched with *P*-values of 0.05 or lower are displayed.

#### Table of transcriptional regulators

The Regulation tab page for genes present in the regulator-target data as targets (6224 genes as of October 2013, including protein-coding, RNA-coding, transposable element genes and pseudogenes) contains a ‘Regulators’ table displaying annotations supporting relationships between these target genes and their regulators. The table header shows the number of regulator genes as well as the number of annotations. The data types shown in the Regulators table are the same as those described previously for the Targets table.

#### Network visualization

To better convey the complex relationships between regulators and targets, the Regulation pages for most genes display a network visualization of regulatory relationships (Figure 3). The network is built using the set of regulatory relationships between all of the targets and regulators of the central gene in the SGD database. We rank the confidence of each regulatory relationship based on the number of different annotations that support it, and show the highest confidence relationships. The network is drawn using Cytoscape.js [http://cytoscape.github.io/cytoscape.js/; (24)], an open-source graph analysis and visualization library. It is fully interactive: users can zoom in and out or move genes around within the network. The data may be filtered to see only targets or only regulators of the central gene, and the user can adjust the cutoff for the number of annotations supporting the displayed relationships to see more or less of the regulatory neighborhood of the central gene.

### Viewing and accessing regulation data in SGD

#### Locus Summary pages

Regulation pages are accessible from the ‘Regulation’ tab at the top of most SGD Locus Summary pages. Additionally, a ‘Regulation’ section of the Locus Summary page displays the numbers of transcriptional regulatory targets and regulators for each gene, which link to the tables of regulation annotations on the Regulation page. This section of the Locus Summary page also contains a gene-specific link to the gene’s page in the YEASTRACT database. If information about the gene is available in the ScerTF [http://stormo.wustl.edu/ScerTF/; (25)], UniProbe [http://the_brain.bwh.harvard.edu/uniprobe/index.php; (26)] or YeTFaSCo [http://yetfasco.ccbr.utoronto.ca/; (6)] databases, links to those resources are also present.

*The YeastMine data warehouse.* Although the Regulation web pages provide a way to view and download regulation data relevant to single genes, YeastMine provides access to regulation data as well as virtually all curated data in SGD (23). This powerful tool allows retrieval of custom sets of data for single or multiple genes. The YeastMine templates (pre-composed queries) available for regulation data are listed in Table 2. The templates ‘Gene (regulator) → Gene (targets)’ and ‘Gene (target) → Gene (regulators)’ retrieve regulation annotations and all data associated with them, including direction of regulation and confidence score when available. YeastMine allows the regulation data to be analyzed further by intersecting them with particular sets of genes, such as those involved in certain pathways or processes, and with other types of data, such as expression levels, protein–protein or genetic interactions or homologs in other species.

**Table 2.** YeastMine template queries for regulation data

Template name	Description
Retrieve all Regulators and their Summary Paragraphs	Retrieve the list of all regulator genes that have Regulation Summary paragraphs, along with the paragraph text and references
Gene (regulator) → Gene (targets)	Start with the gene name or systematic name of a regulator (or a list) and retrieve its target genes, along with evidence (ECO term name and ID), reference and source. For some targets, data on experimental condition, direction of regulation (activated or repressed), *P*-value or false discovery rate are also available.
Gene → Regulation Summary + References	Start with the gene name or systematic name of a regulator (or a list) and retrieve its Regulation Summary paragraph and references for the paragraph.
Gene → PWM-predicted binding sites^a^	Start with the gene name or systematic name of a regulator (or a list) and retrieve the number of its binding sites that are predicted in the genome, either between genes or within genes. Numbers predicted at three different *P*-values are shown.
Gene (target) → Gene (regulators)	Start with the gene name or systematic name of a target (or a list) and retrieve its regulators, along with evidence (ECO term name and ID), reference and source. For some targets, data on experimental condition, direction of regulation (activated or repressed), *P*-value or false discovery rate are also available.
Gene → Protein →Domains	Start with a gene name or systematic name (or a list) and retrieve its domains as determined by InterProScan analysis.
JASPAR Class → Genes	Select the name of a transcription factor class, as defined by the JASPAR database, from the pull-down menu and retrieve all of the *S. cerevisiae* transcription factors in that class.
JASPAR Family → Genes	Select the name of a transcription factor family, as defined by the JASPAR database, from the pull-down menu and retrieve all of the *S. cerevisiae* transcription factors in that family.

All the template queries are categorized as ‘Regulation’ templates in YeastMine (http://yeastmine.yeastgenome.org/yeastmine/begin.do) except for the Gene → Protein → Domains template, which is in the ‘Protein’ category.

^a^To generate the predicted binding site data, genomic matches to the high- and medium-confidence position-weighted matrices (PWMs) from the YeTFaSCo database were located using the FIMO algorithm (20). The fjoin algorithm (27) was used to determine the overlap of matches at different FIMO *P*-value thresholds (*P* < 1e-03, 1e-04 and 1e-05) with either ORF features from the *saccharomyces_ce*re*visiae*.gff file (including Dubious ORFs; available at http://www.yeastgenome.org/download-data/sequence) or with intergenic regions (as downloaded from YeastMine using the ‘Gene-→Upstream intergenic region’ template for all ORFs). Overlap was defined to be at least 1 overlapping basepair.

## DISCUSSION

A more comprehensive picture of *S. cerevisiae* regulatory networks would be useful not only for systems biologists and industrial scientists, but also for researchers seeking to understand individual pathways and processes in yeast and in other organisms. So far, a major obstacle for this goal has been the lack of machine-readable data. For example, Geistlinger and coworkers recently constructed a detailed model of regulation during the diauxic shift, but to do so they needed to manually recurate ∼400 articles. Although the articles had been curated by SGD and YEASTRACT, not enough detail had been captured to construct an accurate network (8).

This project seeks to remedy that situation by capturing a variety of regulation-related data in a controlled format that can be browsed, downloaded, analyzed and visualized. To present researchers with all the available curated data in one convenient location, we have begun to collaborate with the YEASTRACT database (3) to make the annotations from both databases available from the SGD Web site. The YEASTRACT interface continues to provide alternative ways to access and analyze the data, so that the two sites are complementary rather than redundant.

Although our current curation methods capture many of the details of transcriptional regulation, there are still more nuances that can and should be curated in the future. Many regulatory relationships are binary, but others involve multiple regulators—for example, subunits of a regulator complex or TFs that act in concert with other TFs. Regulatory features of the genome such as promoters, TF binding sites and chromatin structure need to be more fully annotated and integrated with other genome annotation.

Transcription is of course only one of many levels at which regulation can occur. Processing, stability or localization of mRNAs; initiation, elongation, or termination of translation; and protein modification, stability, localization or activity are also points of regulation, and most signaling pathways include several of these types in addition to transcriptional regulation. The challenge for the future will be to devise ways to capture all these regulatory relationships and to integrate them into functional models of cellular pathways.

## FUNDING

Funding for open access charge: National Human Genome Research Institute [U41 HG001315].

*Conflict of interest statement*. None declared.
